# Poly[diaqua­bis(μ_3_-1*H*-benzimidazole-5,6-dicarboxyl­ato-κ^4^ *N* ^3^:*O* ^5^,*O* ^5′^:*O* ^6^)bis­(μ_2_-1*H*,3*H*-benzimidazolium-5,6-dicarboxyl­ato-κ^3^ *O* ^5^,*O* ^5′^:*O* ^6^)digadolinium(III)]

**DOI:** 10.1107/S1600536809046819

**Published:** 2009-11-14

**Authors:** Jie-Xuan Huang, Yi-Yi Wu, Chun-De Huang, Qing-Yang Lian, Rong-Hua Zeng

**Affiliations:** a School of Chemistry and Environment, South China Normal University, Guangzhou 510006, People’s Republic of China; b Key Laboratory of Technology of Electrochemical Energy Storage and Power Generation in Guangdong Universities, South China Normal University, Guangzhou 510006, People’s Republic of China

## Abstract

In the title complex, [Gd_2_(C_9_H_4_N_2_O_4_)_2_(C_9_H_5_N_2_O_4_)_2_(H_2_O)_2_]_*n*_, two of the benzimidazole-5,6-dicarboxyl­ate ligands are pro­ton­ated at the imidazole groups. Each Gd^III^ ion is coordinated by six O atoms and one N atom from five ligands and one water mol­ecule, displaying a distorted bicapped trigonal-prismatic geometry. The Gd^III^ ions are linked by the carboxyl­ate groups and imidazole N atoms, forming a layer parallel to (001). These layers are further connected by O—H⋯O and N—H⋯O hydrogen bonds into a three-dimensional supra­molecular network.

## Related literature

For related structures, see: Gao *et al.* (2008 ▶); Lo *et al.* (2007 ▶); Wei *et al.* (2008 ▶); Yao *et al.* (2008 ▶).
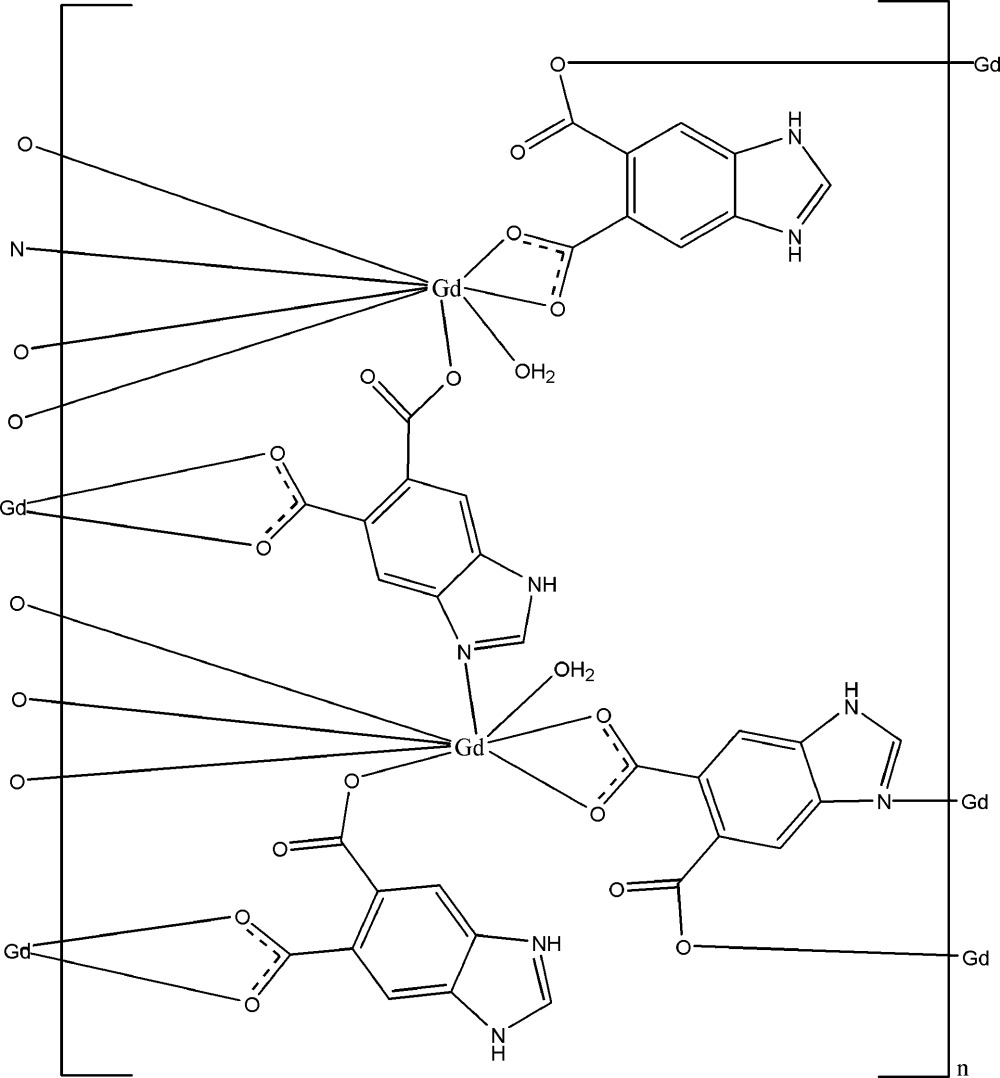


## Experimental

### 

#### Crystal data

[Gd_2_(C_9_H_4_N_2_O_4_)_2_(C_9_H_5_N_2_O_4_)_2_(H_2_O)_2_]
*M* *_r_* = 1169.12
Monoclinic, 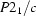

*a* = 18.7856 (11) Å
*b* = 12.7745 (7) Å
*c* = 15.4776 (9) Å
β = 108.010 (1)°
*V* = 3532.3 (3) Å^3^
*Z* = 4
Mo *K*α radiation
μ = 3.82 mm^−1^
*T* = 296 K
0.25 × 0.24 × 0.21 mm

#### Data collection

Bruker APEXII CCD diffractometer
Absorption correction: multi-scan (*SADABS*; Sheldrick, 1996 ▶) *T*_min_ = 0.448, *T*_max_ = 0.501
24827 measured reflections
6338 independent reflections
5929 reflections with *I* > 2σ(*I*)
*R*_int_ = 0.027

#### Refinement

*R*[*F* ^2^ > 2σ(*F* ^2^)] = 0.020
*wR*(*F* ^2^) = 0.049
*S* = 1.08
6338 reflections
595 parameters
13 restraints
H atoms treated by a mixture of independent and constrained refinement
Δρ_max_ = 0.63 e Å^−3^
Δρ_min_ = −0.55 e Å^−3^

### 

Data collection: *APEX2* (Bruker, 2007 ▶); cell refinement: *SAINT* (Bruker, 2007 ▶); data reduction: *SAINT*; program(s) used to solve structure: *SHELXS97* (Sheldrick, 2008 ▶); program(s) used to refine structure: *SHELXL97* (Sheldrick, 2008 ▶); molecular graphics: *DIAMOND* (Brandenburg, 1999 ▶); software used to prepare material for publication: *SHELXTL* (Sheldrick, 2008 ▶).

## Supplementary Material

Crystal structure: contains datablocks I, global. DOI: 10.1107/S1600536809046819/hy2247sup1.cif

Structure factors: contains datablocks I. DOI: 10.1107/S1600536809046819/hy2247Isup2.hkl

Additional supplementary materials:  crystallographic information; 3D view; checkCIF report

## Figures and Tables

**Table 1:** Selected bond lengths (Å)

Gd1—O1	2.338 (2)
Gd1—O2	2.526 (2)
Gd1—O3^i^	2.350 (2)
Gd1—O5^ii^	2.461 (2)
Gd1—O6^ii^	2.499 (2)
Gd1—O8	2.314 (2)
Gd1—N6	2.617 (3)
Gd1—O1*W*	2.374 (2)
Gd2—O9	2.312 (2)
Gd2—O11^iii^	2.539 (2)
Gd2—O12^iii^	2.342 (2)
Gd2—O13^iv^	2.267 (2)
Gd2—O15	2.449 (2)
Gd2—O16	2.520 (2)
Gd2—N1^iii^	2.612 (3)
Gd2—O2*W*	2.384 (2)

Symmetry codes: (i) 
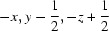
; (ii) 
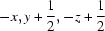
; (iii) 
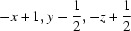
; (iv) 
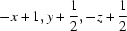
.

**Table 2:** Hydrogen-bond geometry (Å, °)

*D*—H⋯*A*	*D*—H	H⋯*A*	*D*⋯*A*	*D*—H⋯*A*
O1*W*—H1*W*⋯O7^v^	0.84	1.80	2.629 (3)	167
O1*W*—H2*W*⋯O4^i^	0.82	1.92	2.670 (3)	153
O2*W*—H3*W*⋯O10^vi^	0.86	1.74	2.590 (3)	172
O2*W*—H4*WA*⋯O15	0.82	2.14	2.716 (5)	127
O2*W*—H4*WB*⋯O2*W* ^vi^	0.82	1.91	2.723 (4)	170
N2—H2*A*⋯O14^vii^	0.82 (4)	1.94 (4)	2.742 (3)	169 (3)
N3—H3*A*⋯O10^vii^	0.81 (4)	1.94 (4)	2.731 (4)	166 (4)
N4—H4*A*⋯O16	0.84 (4)	1.97 (4)	2.801 (4)	170 (4)
N5—H5*A*⋯O4^v^	0.83 (4)	1.96 (4)	2.772 (3)	165 (4)
N7—H7*A*⋯O5	0.77 (4)	2.08 (4)	2.845 (3)	171 (4)
N8—H8*A*⋯O7^viii^	0.80 (4)	1.95 (4)	2.747 (3)	172 (4)

Symmetry codes: (i) 
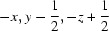
; (v) 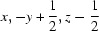; (vi) 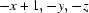; (vii) 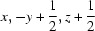; (viii) 
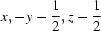
.

**Fig. 1.**
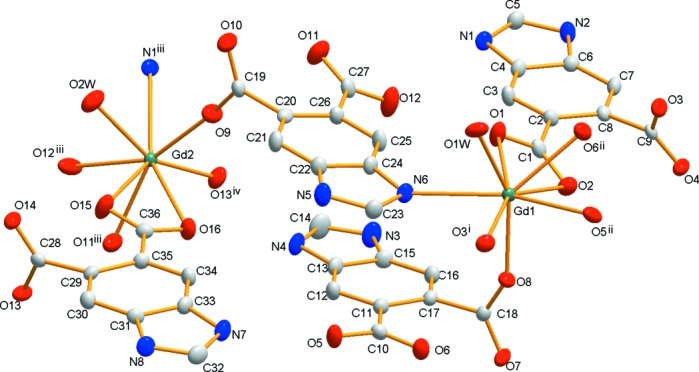
The asymmetric unit of the title compound. Displacement ellipsoids are shown at the 50% probability level. H atoms have been omitted for clarity. [Symmetry codes: (i) -x, -1/2+y, 1/2-z; (ii) -x, 1/2+y, 1/2-z; (iii) 1-x, -1/2+y, 1/2-z; (iv) 1-x, 1/2+y, 1/2-z.]

**Fig. 2.**
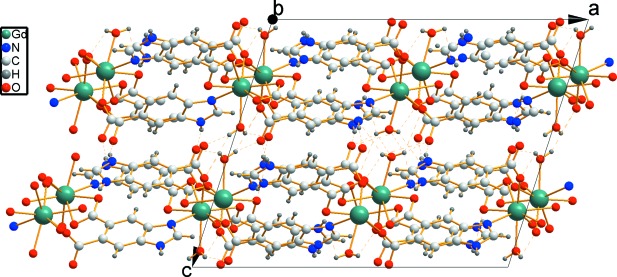
A view of the three-dimensional supramolecular network. Hydrogen bonds are shown as dashed lines.

## supplementary crystallographic information

### Comment

In recent years, research on coordination polymers has made considerable progress in the fields of supramolecular chemistry and crystal engineering, because of their intriguing structural motifs and functional properties, such as molecular adsorption, magnetism and luminescence. In general, the structural motifs of these hybrid compounds are closely related to the geometries of metal centers and the number of coordination sites provided by multidentate ligands. On the other hand, the supramolecular interactions such as hydrogen-bonding, π–π stacking and metallophilic interactions also play the key roles in the recognition process forming final three-dimensional architectures. As a building block, benzimidazole-5,6-dicarboxylic acid is a good ligand with multifunctional coordination sites providing intriguing architectures and topologies (Gao *et al.*, 2008; Lo *et al.*, 2007; Wei *et al.*, 2008; Yao *et al.*, 2008). Recently, we obtained the title coordination polymer, which was synthesized under hydrothermal conditions.

In the title compound (Fig. 1), two of the benzimidazole-5,6-dicarboxylate ligands are protonated at the imidazole groups. Each Gd^III^ ion is eight-coordinated by six O atoms one N atom from five ligands and one water molecule. The coordination geometry can be described as distorted bicapped trigonal prismatic, with Gd—O distances and O—Gd—O angles ranging from 2.267 (2) to 2.539 (2) Å (Table 1) and 52.14 (7) to 156.82 (8)°, respectively. The benzimidazole-5,6-dicarboxylate ligands acting as bridging ligands link the Gd^III^ centers into a layer parallel to the (0 0 1) plane. O—H···O and N—H···O hydrogen bonds (Table 2) connect the layers into a three-dimensional supramolecular motif (Fig. 2). Within the layer, the π–π stacking interactions between neighboring imidazole and benzene rings [centroid–centroid distances = 3.629 (3), 3.755 (4), 3.656 (3) and 3.606 (3) Å] enhance the stability of the crystal structure.

### Experimental

A mixture of Gd_2_O_3_ (0.363 g, 1 mmol), benzimidazole-5,6-dicarboxylic acid (0.206 g, 1 mmol), water (10 ml) in the presence of HClO_4_ (0.039 g, 0.385 mmol) was stirred vigorously for 30 min and then sealed in a Teflon-lined stainless-steel autoclave (20 ml capacity). The autoclave was heated and maintained at 433 K for 3 d, and then cooled to room temperature at 5 K h^-1^. The colorless block crystals were obtained.

### Refinement

Water H atoms were tentatively located in difference Fourier maps and fixed in refinements, with *U*_iso_(H) = 1.5*U*_eq_(O). One of the H atoms of O2W is disordered over two sites (O4WA and O4WB), each with an occupancy factor of 0.5. H atoms on N atoms were tentatively located in difference Fourier maps and refined with distance restraints of N—H = 0.82 (1) Å and with *U*_iso_(H) = 1.2*U*_eq_(N). H atoms attached to C atoms were positioned geometrically and refined as riding, with C—H = 0.93 Å and with *U*_iso_(H) = 1.2*U*_eq_(C).

### Crystal data

**Table:**

[Gd_2_(C_9_H_4_N_2_O_4_)_2_(C_9_H_5_N_2_O_4_)_2_(H_2_O)_2_]	*F*(000) = 2264
*M**_r_* = 1169.12	*D*_x_ = 2.198 Mg m^−^^3^
Monoclinic, *P*2_1_/*c*	Mo *K*α radiation, λ = 0.71073 Å
Hall symbol: -P 2ybc	Cell parameters from 7234 reflections
*a* = 18.7856 (11) Å	θ = 2.6–28.2°
*b* = 12.7745 (7) Å	µ = 3.82 mm^−^^1^
*c* = 15.4776 (9) Å	*T* = 296 K
β = 108.010 (1)°	Block, colorless
*V* = 3532.3 (3) Å^3^	0.25 × 0.24 × 0.21 mm
*Z* = 4	

### Data collection

**Table:**

Bruker APEXII CCD diffractometer	6338 independent reflections
Radiation source: fine-focus sealed tube	5929 reflections with *I* > 2σ(*I*)
graphite	*R*_int_ = 0.027
φ and ω scans	θ_max_ = 25.2°, θ_min_ = 2.0°
Absorption correction: multi-scan (*SADABS*; Sheldrick, 1996)	*h* = −21→22
*T*_min_ = 0.448, *T*_max_ = 0.501	*k* = −15→15
24827 measured reflections	*l* = −18→18

### Refinement

**Table:**

Refinement on *F*^2^	Primary atom site location: structure-invariant direct methods
Least-squares matrix: full	Secondary atom site location: difference Fourier map
*R*[*F*^2^ > 2σ(*F*^2^)] = 0.020	Hydrogen site location: inferred from neighbouring sites
*wR*(*F*^2^) = 0.049	H atoms treated by a mixture of independent and constrained refinement
*S* = 1.08	*w* = 1/[σ^2^(*F*_o_^2^) + (0.021*P*)^2^ + 3.3986*P*] where *P* = (*F*_o_^2^ + 2*F*_c_^2^)/3
6338 reflections	(Δ/σ)_max_ = 0.002
595 parameters	Δρ_max_ = 0.63 e Å^−^^3^
13 restraints	Δρ_min_ = −0.55 e Å^−^^3^

### Fractional atomic coordinates and isotropic or equivalent isotropic displacement parameters (Å^2^)

**Table:**

	*x*	*y*	*z*	*U*_iso_*/*U*_eq_	Occ. (<1)
Gd1	0.028568 (8)	0.296570 (11)	0.225244 (9)	0.01343 (5)	
Gd2	0.524307 (8)	−0.054993 (10)	0.209686 (9)	0.01317 (5)	
N6	0.10953 (14)	0.1586 (2)	0.17264 (18)	0.0208 (6)	
N5	0.12241 (15)	0.0250 (2)	0.08627 (19)	0.0251 (6)	
H5A	0.111 (2)	−0.029 (3)	0.056 (2)	0.030*	
C23	0.07832 (18)	0.0751 (2)	0.1265 (2)	0.0218 (7)	
H23	0.0302	0.0529	0.1222	0.026*	
C25	0.24026 (17)	0.2316 (2)	0.1949 (2)	0.0220 (7)	
H25	0.2367	0.2863	0.2331	0.026*	
C21	0.25303 (17)	0.0637 (2)	0.0817 (2)	0.0215 (7)	
H21	0.2569	0.0077	0.0449	0.026*	
C24	0.18074 (16)	0.1631 (2)	0.1607 (2)	0.0181 (6)	
C22	0.18850 (17)	0.0794 (2)	0.1059 (2)	0.0197 (6)	
C20	0.31133 (16)	0.1340 (2)	0.11398 (19)	0.0166 (6)	
C26	0.30518 (17)	0.2172 (2)	0.1711 (2)	0.0178 (6)	
N3	0.36219 (16)	0.1575 (2)	0.4264 (2)	0.0312 (7)	
H3A	0.375 (2)	0.212 (3)	0.453 (3)	0.037*	
C13	0.28840 (17)	0.0294 (2)	0.3540 (2)	0.0199 (7)	
N4	0.35934 (15)	0.0200 (2)	0.3445 (2)	0.0256 (6)	
H4A	0.372 (2)	−0.029 (3)	0.316 (2)	0.031*	
C15	0.29033 (17)	0.1190 (2)	0.4058 (2)	0.0195 (6)	
C14	0.40105 (19)	0.0970 (3)	0.3890 (2)	0.0334 (8)	
H14	0.4511	0.1073	0.3934	0.040*	
C27	0.36712 (17)	0.2947 (2)	0.2069 (2)	0.0207 (7)	
C19	0.38033 (17)	0.1168 (2)	0.0861 (2)	0.0181 (6)	
O11	0.41746 (14)	0.30726 (19)	0.17120 (17)	0.0342 (6)	
O10	0.37957 (14)	0.15189 (19)	0.01034 (16)	0.0338 (6)	
C17	0.16278 (17)	0.0978 (2)	0.39339 (19)	0.0163 (6)	
C12	0.22389 (17)	−0.0299 (2)	0.3229 (2)	0.0206 (7)	
H12	0.2230	−0.0912	0.2902	0.025*	
C16	0.22774 (17)	0.1551 (2)	0.4263 (2)	0.0199 (7)	
H16	0.2295	0.2153	0.4607	0.024*	
C11	0.16054 (17)	0.0050 (2)	0.3421 (2)	0.0177 (6)	
C10	0.08996 (17)	−0.0570 (2)	0.3073 (2)	0.0185 (7)	
C18	0.09517 (16)	0.1407 (2)	0.41501 (19)	0.0161 (6)	
O8	0.05302 (12)	0.20217 (15)	0.35883 (14)	0.0207 (5)	
O7	0.08767 (13)	0.11675 (18)	0.48980 (14)	0.0269 (5)	
O5	0.08938 (12)	−0.13723 (16)	0.25772 (16)	0.0266 (5)	
O6	0.03295 (12)	−0.03134 (16)	0.32721 (15)	0.0246 (5)	
O12	0.36722 (13)	0.3474 (2)	0.27665 (18)	0.0391 (7)	
C36	0.40339 (17)	−0.1991 (2)	0.1826 (2)	0.0185 (6)	
C33	0.20968 (17)	−0.2999 (2)	0.1476 (2)	0.0192 (6)	
C35	0.33593 (17)	−0.2677 (2)	0.1548 (2)	0.0175 (6)	
C34	0.27150 (17)	−0.2345 (2)	0.1727 (2)	0.0200 (7)	
H34	0.2699	−0.1705	0.2006	0.024*	
O16	0.40712 (12)	−0.12307 (16)	0.23682 (15)	0.0246 (5)	
O15	0.45572 (13)	−0.21597 (18)	0.15068 (17)	0.0320 (6)	
O9	0.43302 (13)	0.06471 (18)	0.13734 (16)	0.0294 (5)	
N7	0.14022 (15)	−0.2953 (2)	0.16138 (19)	0.0242 (6)	
H7A	0.127 (2)	−0.248 (3)	0.184 (2)	0.029*	
C28	0.40817 (17)	−0.4085 (2)	0.09601 (19)	0.0163 (6)	
C29	0.33774 (16)	−0.3652 (2)	0.11096 (19)	0.0159 (6)	
C32	0.10256 (17)	−0.3811 (3)	0.1275 (2)	0.0233 (7)	
H32	0.0547	−0.3968	0.1292	0.028*	
O14	0.41716 (12)	−0.40122 (17)	0.02045 (14)	0.0237 (5)	
N8	0.14248 (15)	−0.4412 (2)	0.09098 (19)	0.0219 (6)	
H8A	0.126 (2)	−0.495 (3)	0.066 (2)	0.026*	
C31	0.21123 (16)	−0.3943 (2)	0.1031 (2)	0.0168 (6)	
C30	0.27505 (17)	−0.4281 (2)	0.0840 (2)	0.0192 (6)	
H30	0.2755	−0.4911	0.0539	0.023*	
C1	0.13784 (17)	0.4457 (2)	0.3037 (2)	0.0188 (7)	
C2	0.19826 (17)	0.5268 (2)	0.3348 (2)	0.0176 (6)	
O2	0.09162 (12)	0.42965 (17)	0.34523 (15)	0.0249 (5)	
O1	0.13579 (12)	0.39355 (17)	0.23230 (16)	0.0287 (5)	
C8	0.18820 (16)	0.6163 (2)	0.38430 (19)	0.0158 (6)	
C7	0.24484 (17)	0.6887 (2)	0.4144 (2)	0.0185 (6)	
H7	0.2389	0.7474	0.4471	0.022*	
C4	0.32222 (16)	0.5828 (2)	0.3467 (2)	0.0175 (6)	
C6	0.31138 (16)	0.6714 (2)	0.39437 (19)	0.0172 (6)	
C3	0.26477 (16)	0.5098 (2)	0.3167 (2)	0.0193 (7)	
H3	0.2711	0.4506	0.2849	0.023*	
N1	0.39340 (14)	0.5868 (2)	0.33531 (17)	0.0190 (5)	
N2	0.37599 (15)	0.7294 (2)	0.41004 (18)	0.0217 (6)	
H2A	0.385 (2)	0.785 (3)	0.437 (2)	0.026*	
C5	0.42138 (17)	0.6759 (2)	0.3735 (2)	0.0208 (7)	
H5	0.4685	0.6999	0.3752	0.025*	
C9	0.11320 (17)	0.6424 (2)	0.3956 (2)	0.0165 (6)	
O4	0.10804 (12)	0.64817 (17)	0.47426 (14)	0.0242 (5)	
O13	0.45307 (12)	−0.45613 (17)	0.16304 (14)	0.0247 (5)	
O3	0.06077 (11)	0.66278 (16)	0.32358 (14)	0.0203 (5)	
O1W	−0.00880 (13)	0.30447 (17)	0.06416 (14)	0.0264 (5)	
H1W	0.0184	0.3242	0.0331	0.040*	
H2W	−0.0314	0.2519	0.0407	0.040*	
O2W	0.51762 (14)	−0.0852 (2)	0.05540 (15)	0.0346 (6)	
H3W	0.5544	−0.1080	0.0390	0.052*	
H4WA	0.4879	−0.1341	0.0480	0.052*	0.50
H4WB	0.5025	−0.0331	0.0237	0.052*	0.50

### Atomic displacement parameters (Å^2^)

**Table:**

	*U*^11^	*U*^22^	*U*^33^	*U*^12^	*U*^13^	*U*^23^
Gd1	0.01015 (8)	0.01374 (8)	0.01696 (8)	−0.00059 (5)	0.00501 (6)	0.00056 (5)
Gd2	0.01062 (8)	0.01412 (8)	0.01541 (8)	−0.00007 (5)	0.00497 (6)	−0.00022 (5)
N6	0.0124 (13)	0.0236 (14)	0.0276 (14)	−0.0025 (11)	0.0079 (11)	−0.0044 (11)
N5	0.0183 (15)	0.0242 (14)	0.0323 (16)	−0.0062 (12)	0.0071 (12)	−0.0130 (12)
C23	0.0146 (16)	0.0244 (16)	0.0278 (17)	−0.0023 (13)	0.0084 (14)	−0.0022 (13)
C25	0.0183 (17)	0.0186 (15)	0.0294 (17)	−0.0016 (13)	0.0077 (14)	−0.0101 (13)
C21	0.0188 (17)	0.0214 (16)	0.0252 (17)	−0.0004 (13)	0.0080 (14)	−0.0090 (13)
C24	0.0115 (15)	0.0204 (15)	0.0233 (16)	0.0007 (12)	0.0067 (12)	−0.0028 (12)
C22	0.0152 (16)	0.0202 (15)	0.0232 (16)	−0.0026 (12)	0.0052 (13)	−0.0044 (13)
C20	0.0123 (15)	0.0210 (15)	0.0155 (14)	0.0035 (12)	0.0029 (12)	0.0036 (12)
C26	0.0141 (16)	0.0166 (15)	0.0235 (16)	−0.0007 (12)	0.0067 (13)	−0.0023 (12)
N3	0.0182 (16)	0.0345 (17)	0.0425 (18)	−0.0092 (13)	0.0116 (13)	−0.0153 (14)
C13	0.0139 (16)	0.0242 (16)	0.0230 (16)	0.0032 (13)	0.0077 (13)	−0.0003 (13)
N4	0.0163 (15)	0.0311 (16)	0.0328 (16)	0.0008 (12)	0.0126 (12)	−0.0051 (13)
C15	0.0126 (16)	0.0225 (16)	0.0221 (16)	−0.0037 (12)	0.0036 (13)	−0.0016 (13)
C14	0.0156 (18)	0.043 (2)	0.044 (2)	−0.0055 (15)	0.0122 (16)	−0.0085 (17)
C27	0.0136 (16)	0.0185 (15)	0.0300 (18)	0.0017 (12)	0.0068 (14)	−0.0032 (13)
C19	0.0158 (16)	0.0151 (14)	0.0239 (16)	0.0016 (12)	0.0066 (13)	−0.0024 (12)
O11	0.0307 (14)	0.0418 (15)	0.0373 (14)	−0.0177 (11)	0.0211 (12)	−0.0165 (11)
O10	0.0385 (15)	0.0404 (14)	0.0280 (13)	0.0139 (12)	0.0184 (11)	0.0108 (11)
C17	0.0162 (16)	0.0181 (15)	0.0153 (14)	0.0015 (12)	0.0059 (12)	0.0001 (12)
C12	0.0199 (17)	0.0171 (15)	0.0272 (17)	−0.0006 (13)	0.0108 (14)	−0.0045 (13)
C16	0.0193 (17)	0.0188 (15)	0.0232 (16)	−0.0021 (12)	0.0090 (13)	−0.0043 (13)
C11	0.0144 (16)	0.0189 (15)	0.0205 (15)	−0.0001 (12)	0.0065 (13)	0.0003 (12)
C10	0.0178 (17)	0.0150 (15)	0.0227 (16)	−0.0015 (12)	0.0062 (13)	0.0010 (12)
C18	0.0144 (16)	0.0163 (14)	0.0185 (15)	−0.0019 (12)	0.0062 (12)	−0.0018 (12)
O8	0.0202 (12)	0.0202 (11)	0.0221 (11)	0.0063 (9)	0.0070 (9)	0.0064 (9)
O7	0.0288 (13)	0.0343 (13)	0.0225 (12)	0.0150 (10)	0.0151 (10)	0.0088 (10)
O5	0.0197 (12)	0.0208 (11)	0.0433 (14)	−0.0069 (9)	0.0155 (11)	−0.0133 (10)
O6	0.0162 (12)	0.0223 (11)	0.0380 (13)	−0.0038 (9)	0.0124 (10)	−0.0065 (10)
O12	0.0252 (14)	0.0405 (15)	0.0588 (17)	−0.0157 (11)	0.0233 (13)	−0.0302 (13)
C36	0.0173 (17)	0.0195 (15)	0.0186 (15)	−0.0020 (12)	0.0054 (13)	0.0015 (12)
C33	0.0130 (16)	0.0244 (16)	0.0213 (16)	0.0017 (12)	0.0066 (13)	−0.0002 (13)
C35	0.0150 (16)	0.0185 (15)	0.0189 (15)	−0.0016 (12)	0.0053 (13)	−0.0006 (12)
C34	0.0199 (17)	0.0160 (15)	0.0249 (16)	−0.0002 (12)	0.0083 (14)	−0.0042 (12)
O16	0.0219 (12)	0.0203 (11)	0.0351 (13)	−0.0064 (9)	0.0136 (10)	−0.0118 (10)
O15	0.0281 (14)	0.0341 (13)	0.0422 (15)	−0.0139 (11)	0.0232 (12)	−0.0182 (11)
O9	0.0184 (13)	0.0341 (13)	0.0370 (14)	0.0092 (10)	0.0106 (11)	0.0128 (11)
N7	0.0180 (15)	0.0282 (16)	0.0287 (16)	0.0006 (12)	0.0104 (12)	−0.0061 (12)
C28	0.0180 (16)	0.0127 (14)	0.0181 (15)	−0.0019 (12)	0.0052 (13)	−0.0002 (12)
C29	0.0155 (16)	0.0190 (15)	0.0146 (14)	−0.0001 (12)	0.0069 (12)	0.0003 (12)
C32	0.0122 (16)	0.0313 (18)	0.0259 (17)	−0.0018 (13)	0.0053 (13)	0.0008 (14)
O14	0.0258 (13)	0.0289 (12)	0.0184 (11)	0.0076 (10)	0.0097 (10)	0.0039 (9)
N8	0.0121 (14)	0.0244 (15)	0.0276 (15)	−0.0051 (11)	0.0039 (12)	−0.0046 (12)
C31	0.0120 (15)	0.0181 (15)	0.0187 (15)	−0.0029 (12)	0.0024 (12)	0.0000 (12)
C30	0.0177 (17)	0.0180 (15)	0.0216 (16)	−0.0004 (12)	0.0055 (13)	−0.0032 (12)
C1	0.0121 (16)	0.0158 (15)	0.0268 (17)	0.0017 (12)	0.0033 (13)	−0.0022 (12)
C2	0.0141 (16)	0.0158 (15)	0.0229 (16)	0.0003 (12)	0.0057 (13)	0.0001 (12)
O2	0.0235 (13)	0.0260 (12)	0.0282 (12)	−0.0070 (9)	0.0122 (10)	−0.0035 (10)
O1	0.0207 (13)	0.0270 (12)	0.0441 (14)	−0.0100 (10)	0.0186 (11)	−0.0172 (11)
C8	0.0141 (16)	0.0158 (14)	0.0165 (14)	0.0027 (12)	0.0033 (12)	0.0010 (11)
C7	0.0179 (16)	0.0194 (15)	0.0186 (15)	0.0029 (12)	0.0064 (13)	−0.0032 (12)
C4	0.0117 (16)	0.0212 (15)	0.0188 (15)	0.0003 (12)	0.0035 (12)	−0.0029 (12)
C6	0.0144 (16)	0.0192 (15)	0.0166 (15)	−0.0001 (12)	0.0029 (12)	−0.0004 (12)
C3	0.0138 (16)	0.0159 (15)	0.0276 (17)	0.0003 (12)	0.0057 (13)	−0.0060 (12)
N1	0.0104 (13)	0.0228 (13)	0.0229 (13)	−0.0027 (10)	0.0038 (11)	−0.0038 (11)
N2	0.0166 (14)	0.0196 (14)	0.0282 (15)	−0.0033 (11)	0.0060 (12)	−0.0092 (12)
C5	0.0154 (16)	0.0241 (16)	0.0240 (16)	−0.0015 (13)	0.0075 (13)	−0.0015 (13)
C9	0.0161 (16)	0.0110 (14)	0.0235 (16)	0.0036 (11)	0.0077 (13)	0.0013 (12)
O4	0.0261 (13)	0.0288 (12)	0.0214 (11)	0.0088 (10)	0.0128 (10)	0.0055 (9)
O13	0.0208 (12)	0.0322 (13)	0.0224 (12)	0.0071 (10)	0.0084 (10)	0.0112 (10)
O3	0.0131 (11)	0.0248 (11)	0.0218 (11)	0.0054 (9)	0.0038 (9)	0.0013 (9)
O1W	0.0313 (14)	0.0279 (12)	0.0216 (12)	−0.0129 (10)	0.0108 (10)	0.0001 (9)
O2W	0.0307 (14)	0.0532 (16)	0.0215 (12)	−0.0001 (12)	0.0108 (11)	−0.0028 (11)

### Geometric parameters (Å, °)

**Table:**

Gd1—O1	2.338 (2)	C16—H16	0.9300
Gd1—O2	2.526 (2)	C11—C10	1.495 (4)
Gd1—O3^i^	2.350 (2)	C10—O6	1.247 (4)
Gd1—O5^ii^	2.461 (2)	C10—O5	1.278 (4)
Gd1—O6^ii^	2.499 (2)	C18—O7	1.247 (4)
Gd1—O8	2.314 (2)	C18—O8	1.255 (3)
Gd1—N6	2.617 (3)	C36—O15	1.248 (4)
Gd1—O1W	2.374 (2)	C36—O16	1.271 (4)
Gd2—O9	2.312 (2)	C36—C35	1.491 (4)
Gd2—O11^iii^	2.539 (2)	C33—C34	1.385 (4)
Gd2—O12^iii^	2.342 (2)	C33—N7	1.387 (4)
Gd2—O13^iv^	2.267 (2)	C33—C31	1.393 (4)
Gd2—O15	2.449 (2)	C35—C34	1.388 (4)
Gd2—O16	2.520 (2)	C35—C29	1.424 (4)
Gd2—N1^iii^	2.612 (3)	C34—H34	0.9300
Gd2—O2W	2.384 (2)	N7—C32	1.323 (4)
N6—C23	1.316 (4)	N7—H7A	0.77 (4)
N6—C24	1.407 (4)	C28—O14	1.236 (4)
N5—C23	1.343 (4)	C28—O13	1.272 (4)
N5—C22	1.372 (4)	C28—C29	1.516 (4)
N5—H5A	0.83 (4)	C29—C30	1.380 (4)
C23—H23	0.9300	C32—N8	1.316 (4)
C25—C24	1.389 (4)	C32—H32	0.9300
C25—C26	1.391 (4)	N8—C31	1.382 (4)
C25—H25	0.9300	N8—H8A	0.80 (4)
C21—C20	1.385 (4)	C31—C30	1.390 (4)
C21—C22	1.390 (4)	C30—H30	0.9300
C21—H21	0.9300	C1—O2	1.247 (4)
C24—C22	1.400 (4)	C1—O1	1.281 (4)
C20—C26	1.411 (4)	C1—C2	1.502 (4)
C20—C19	1.504 (4)	C2—C3	1.380 (4)
C26—C27	1.498 (4)	C2—C8	1.420 (4)
N3—C14	1.314 (4)	C8—C7	1.378 (4)
N3—C15	1.378 (4)	C8—C9	1.510 (4)
N3—H3A	0.81 (4)	C7—C6	1.396 (4)
C13—C12	1.383 (4)	C7—H7	0.9300
C13—N4	1.391 (4)	C4—C3	1.392 (4)
C13—C15	1.392 (4)	C4—C6	1.400 (4)
N4—C14	1.313 (4)	C4—N1	1.403 (4)
N4—H4A	0.84 (4)	C6—N2	1.378 (4)
C15—C16	1.387 (4)	C3—H3	0.9300
C14—H14	0.9300	N1—C5	1.315 (4)
C27—O11	1.245 (4)	N2—C5	1.345 (4)
C27—O12	1.270 (4)	N2—H2A	0.82 (4)
C19—O10	1.251 (4)	C5—H5	0.9300
C19—O9	1.251 (4)	C9—O4	1.252 (4)
O11—Gd2^iv^	2.539 (2)	C9—O3	1.266 (4)
C17—C16	1.378 (4)	O1W—H1W	0.84
C17—C11	1.420 (4)	O1W—H2W	0.82
C17—C18	1.513 (4)	O2W—H3W	0.86
C12—C11	1.386 (4)	O2W—H4WA	0.82
C12—H12	0.9300	O2W—H4WB	0.82
			
O8—Gd1—O1	107.71 (8)	N3—C14—H14	124.8
O8—Gd1—O3^i^	80.39 (7)	O11—C27—O12	120.5 (3)
O1—Gd1—O3^i^	156.84 (7)	O11—C27—C26	122.0 (3)
O8—Gd1—O1W	150.83 (7)	O12—C27—C26	117.5 (3)
O1—Gd1—O1W	89.84 (8)	O11—C27—Gd2^iv^	64.70 (17)
O3^i^—Gd1—O1W	75.13 (7)	O12—C27—Gd2^iv^	55.80 (16)
O8—Gd1—O5^ii^	91.14 (7)	C26—C27—Gd2^iv^	173.3 (2)
O1—Gd1—O5^ii^	127.16 (7)	O10—C19—O9	124.2 (3)
O3^i^—Gd1—O5^ii^	72.98 (7)	O10—C19—C20	117.1 (3)
O1W—Gd1—O5^ii^	96.56 (8)	O9—C19—C20	118.6 (3)
O8—Gd1—O6^ii^	133.85 (7)	C27—O11—Gd2^iv^	88.98 (18)
O1—Gd1—O6^ii^	81.62 (8)	C16—C17—C11	121.3 (3)
O3^i^—Gd1—O6^ii^	108.82 (7)	C16—C17—C18	115.8 (3)
O1W—Gd1—O6^ii^	70.40 (8)	C11—C17—C18	123.0 (3)
O5^ii^—Gd1—O6^ii^	52.51 (7)	C13—C12—C11	117.9 (3)
O8—Gd1—O2	77.02 (7)	C13—C12—H12	121.1
O1—Gd1—O2	53.69 (7)	C11—C12—H12	121.1
O3^i^—Gd1—O2	148.38 (7)	C17—C16—C15	117.1 (3)
O1W—Gd1—O2	131.50 (7)	C17—C16—H16	121.4
O5^ii^—Gd1—O2	85.58 (8)	C15—C16—H16	121.4
O6^ii^—Gd1—O2	73.23 (7)	C12—C11—C17	120.6 (3)
O8—Gd1—N6	87.31 (8)	C12—C11—C10	118.5 (3)
O1—Gd1—N6	78.24 (8)	C17—C11—C10	120.9 (3)
O3^i^—Gd1—N6	80.57 (8)	O6—C10—O5	120.7 (3)
O1W—Gd1—N6	73.35 (8)	O6—C10—C11	120.3 (3)
O5^ii^—Gd1—N6	153.38 (8)	O5—C10—C11	118.9 (3)
O6^ii^—Gd1—N6	138.26 (8)	O6—C10—Gd1^i^	61.93 (15)
O2—Gd1—N6	119.76 (8)	O5—C10—Gd1^i^	60.23 (15)
O8—Gd1—C10^ii^	115.53 (8)	C11—C10—Gd1^i^	167.3 (2)
O1—Gd1—C10^ii^	106.09 (8)	O7—C18—O8	124.7 (3)
O3^i^—Gd1—C10^ii^	88.80 (8)	O7—C18—C17	117.4 (3)
O1W—Gd1—C10^ii^	79.89 (8)	O8—C18—C17	117.8 (3)
O5^ii^—Gd1—C10^ii^	26.78 (8)	C18—O8—Gd1	148.6 (2)
O6^ii^—Gd1—C10^ii^	26.11 (8)	C10—O5—Gd1^i^	92.99 (18)
O2—Gd1—C10^ii^	81.39 (8)	C10—O6—Gd1^i^	91.96 (17)
N6—Gd1—C10^ii^	152.92 (8)	C27—O12—Gd2^iv^	97.54 (19)
O13^iv^—Gd2—O9	87.94 (8)	O15—C36—O16	120.3 (3)
O13^iv^—Gd2—O12^iii^	107.11 (9)	O15—C36—C35	119.2 (3)
O9—Gd2—O12^iii^	152.90 (8)	O16—C36—C35	120.5 (3)
O13^iv^—Gd2—O2W	153.97 (8)	C34—C33—N7	132.7 (3)
O9—Gd2—O2W	79.66 (9)	C34—C33—C31	121.1 (3)
O12^iii^—Gd2—O2W	77.24 (9)	N7—C33—C31	106.1 (3)
O13^iv^—Gd2—O15	136.70 (8)	C34—C35—C29	120.9 (3)
O9—Gd2—O15	98.56 (8)	C34—C35—C36	118.6 (3)
O12^iii^—Gd2—O15	85.94 (9)	C29—C35—C36	120.6 (3)
O2W—Gd2—O15	68.38 (8)	C33—C34—C35	117.8 (3)
O13^iv^—Gd2—O16	89.20 (7)	C33—C34—H34	121.1
O9—Gd2—O16	75.74 (8)	C35—C34—H34	121.1
O12^iii^—Gd2—O16	125.56 (8)	C36—O16—Gd2	91.50 (18)
O2W—Gd2—O16	109.44 (8)	C36—O15—Gd2	95.47 (18)
O15—Gd2—O16	52.14 (7)	C19—O9—Gd2	168.9 (2)
O13^iv^—Gd2—O11^iii^	80.34 (8)	C32—N7—C33	108.5 (3)
O9—Gd2—O11^iii^	153.99 (8)	C32—N7—H7A	129 (3)
O12^iii^—Gd2—O11^iii^	52.97 (8)	C33—N7—H7A	123 (3)
O2W—Gd2—O11^iii^	119.51 (8)	O14—C28—O13	123.9 (3)
O15—Gd2—O11^iii^	75.38 (9)	O14—C28—C29	119.9 (3)
O16—Gd2—O11^iii^	80.92 (8)	O13—C28—C29	116.1 (3)
O13^iv^—Gd2—N1^iii^	82.99 (8)	C30—C29—C35	120.7 (3)
O9—Gd2—N1^iii^	79.84 (8)	C30—C29—C28	116.4 (3)
O12^iii^—Gd2—N1^iii^	79.83 (8)	C35—C29—C28	122.8 (3)
O2W—Gd2—N1^iii^	72.39 (8)	N8—C32—N7	110.4 (3)
O15—Gd2—N1^iii^	140.32 (8)	N8—C32—H32	124.8
O16—Gd2—N1^iii^	154.59 (7)	N7—C32—H32	124.8
O11^iii^—Gd2—N1^iii^	121.11 (8)	C32—N8—C31	108.8 (3)
O13^iv^—Gd2—C27^iii^	93.85 (9)	C32—N8—H8A	122 (3)
O9—Gd2—C27^iii^	178.09 (9)	C31—N8—H8A	130 (3)
O12^iii^—Gd2—C27^iii^	26.65 (9)	N8—C31—C30	131.9 (3)
O2W—Gd2—C27^iii^	98.94 (9)	N8—C31—C33	106.3 (3)
O15—Gd2—C27^iii^	79.68 (9)	C30—C31—C33	121.7 (3)
O16—Gd2—C27^iii^	103.62 (8)	C29—C30—C31	117.8 (3)
O11^iii^—Gd2—C27^iii^	26.32 (8)	C29—C30—H30	121.1
N1^iii^—Gd2—C27^iii^	101.01 (8)	C31—C30—H30	121.1
C23—N6—C24	104.1 (3)	O2—C1—O1	121.3 (3)
C23—N6—Gd1	120.7 (2)	O2—C1—C2	121.7 (3)
C24—N6—Gd1	132.91 (19)	O1—C1—C2	117.0 (3)
C23—N5—C22	107.6 (3)	C3—C2—C8	121.1 (3)
C23—N5—H5A	126 (3)	C3—C2—C1	117.9 (3)
C22—N5—H5A	127 (3)	C8—C2—C1	121.0 (3)
N6—C23—N5	113.7 (3)	C1—O2—Gd1	88.55 (18)
N6—C23—H23	123.1	C1—O1—Gd1	96.42 (18)
N5—C23—H23	123.1	C7—C8—C2	120.3 (3)
C24—C25—C26	119.0 (3)	C7—C8—C9	117.6 (3)
C24—C25—H25	120.5	C2—C8—C9	121.6 (3)
C26—C25—H25	120.5	C8—C7—C6	118.0 (3)
C20—C21—C22	117.9 (3)	C8—C7—H7	121.0
C20—C21—H21	121.1	C6—C7—H7	121.0
C22—C21—H21	121.1	C3—C4—C6	119.5 (3)
C25—C24—C22	119.3 (3)	C3—C4—N1	130.8 (3)
C25—C24—N6	131.4 (3)	C6—C4—N1	109.6 (3)
C22—C24—N6	109.2 (3)	N2—C6—C7	132.6 (3)
N5—C22—C21	132.2 (3)	N2—C6—C4	105.3 (3)
N5—C22—C24	105.3 (3)	C7—C6—C4	122.1 (3)
C21—C22—C24	122.4 (3)	C2—C3—C4	118.9 (3)
C21—C20—C26	120.4 (3)	C2—C3—H3	120.5
C21—C20—C19	117.2 (3)	C4—C3—H3	120.5
C26—C20—C19	122.3 (3)	C5—N1—C4	103.9 (2)
C25—C26—C20	120.9 (3)	C5—N1—Gd2^iv^	122.2 (2)
C25—C26—C27	117.3 (3)	C4—N1—Gd2^iv^	132.34 (19)
C20—C26—C27	121.7 (3)	C5—N2—C6	106.9 (3)
C14—N3—C15	109.0 (3)	C5—N2—H2A	127 (3)
C14—N3—H3A	128 (3)	C6—N2—H2A	126 (3)
C15—N3—H3A	123 (3)	N1—C5—N2	114.3 (3)
C12—C13—N4	132.9 (3)	N1—C5—H5	122.9
C12—C13—C15	121.0 (3)	N2—C5—H5	122.9
N4—C13—C15	106.1 (3)	O4—C9—O3	124.8 (3)
C14—N4—C13	108.4 (3)	O4—C9—C8	118.8 (3)
C14—N4—H4A	127 (3)	O3—C9—C8	116.3 (3)
C13—N4—H4A	124 (3)	C28—O13—Gd2^iii^	151.0 (2)
N3—C15—C16	131.9 (3)	C9—O3—Gd1^ii^	135.77 (18)
N3—C15—C13	106.0 (3)	H1W—O1W—H2W	108
C16—C15—C13	122.1 (3)	H3W—O2W—H4WA	106
N4—C14—N3	110.4 (3)	H3W—O2W—H4WB	105
N4—C14—H14	124.8		

Symmetry codes: (i) −*x*, *y*−1/2, −*z*+1/2; (ii) −*x*, *y*+1/2, −*z*+1/2; (iii) −*x*+1, *y*−1/2, −*z*+1/2; (iv) −*x*+1, *y*+1/2, −*z*+1/2.

### Hydrogen-bond geometry (Å, °)

**Table:**

*D*—H···*A*	*D*—H	H···*A*	*D*···*A*	*D*—H···*A*
O1W—H1W···O7^v^	0.84	1.80	2.629 (3)	167
O1W—H2W···O4^i^	0.82	1.92	2.670 (3)	153
O2W—H3W···O10^vi^	0.86	1.74	2.590 (3)	172
O2W—H4WA···O15	0.82	2.14	2.716 (5)	127
O2W—H4WB···O2W^vi^	0.82	1.91	2.723 (4)	170
N2—H2A···O14^vii^	0.82 (4)	1.94 (4)	2.742 (3)	169 (3)
N3—H3A···O10^vii^	0.81 (4)	1.94 (4)	2.731 (4)	166 (4)
N4—H4A···O16	0.84 (4)	1.97 (4)	2.801 (4)	170 (4)
N5—H5A···O4^v^	0.83 (4)	1.96 (4)	2.772 (3)	165 (4)
N7—H7A···O5	0.77 (4)	2.08 (4)	2.845 (3)	171 (4)
N8—H8A···O7^viii^	0.80 (4)	1.95 (4)	2.747 (3)	172 (4)

Symmetry codes: (v) *x*, −*y*+1/2, *z*−1/2; (i) −*x*, *y*−1/2, −*z*+1/2; (vi) −*x*+1, −*y*, −*z*; (vii) *x*, −*y*+1/2, *z*+1/2; (viii) *x*, −*y*−1/2, *z*−1/2.
